# Supplementary material for: Maximising the Use of Scarce qPCR Master Mixes
Source: Int J Mol Sci. 2022 Jul 30;23(15):8486. doi: 10.3390/ijms23158486 (PMC9368830; doi:10.3390/ijms23158486)
Supplement: Supplementary file 1 [file ijms-23-08486-s001.zip › ijms-1806405-supplementary.pdf]

Maximising the use of scarce qPCR master mixes

Supplementary Figures

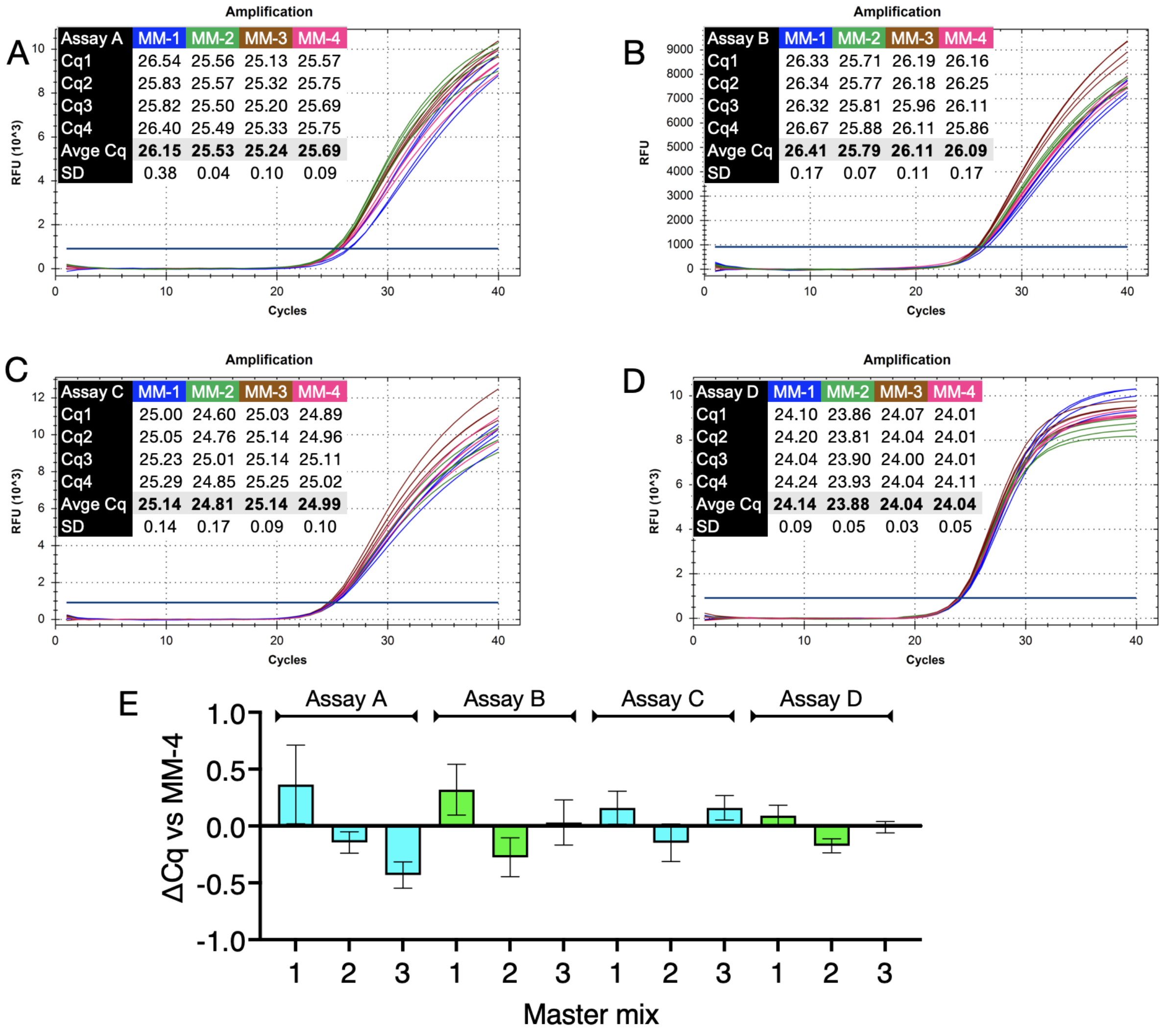

Figure S1. Repeat amplification by MM-1 to MM-4 using modified PCR conditions (2 min activation at 95°C, followed by 40 cycles of 5 second at 95°C denaturation and 10 seconds at 60°C annealing/polymerisation steps). MM-1 (blue), MM-2 (green), MM-3 (brown), MM-4 (pink). Assays A and D were detected by an LNA probe, assays B and C by a DNA probe. **A.** Amplification plots and Cq values assay A (437 bp). **B.** Amplification plots and Cq values assay B (348 bp). **C.** Amplification plots and Cq values assay C (234 bp). **D.** Amplification plots and Cq values assay D (153 bp). **E.** ΔCq values (±SD) for past expiry date MM-1 to 3 relative to the current MM-4.

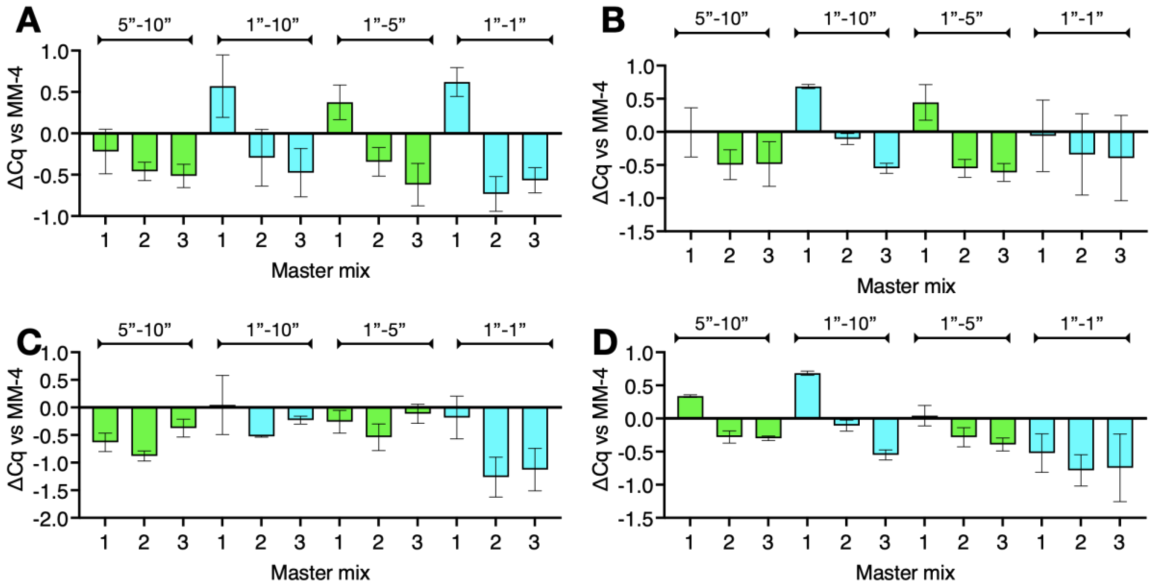

Figure S2. Effect of reducing PCR cycle times on master mix performance. Bars from left to right show ΔCq values (±SD) for "past expiry date" MM-1, MM-2 and MM-3 relative to the current MM-4 recorded using the following protocol: (i) 5 second denaturation/10 seconds polymerisation, (ii) 1 second denaturation/10 seconds polymerisation, (iii) 1 second denaturation/5 second polymerisation and (iv) 1 second denaturation/1 second polymerisation. **A.** Assay A. **B.** Assay B. **C.** Assay A. **D.** Assay D.

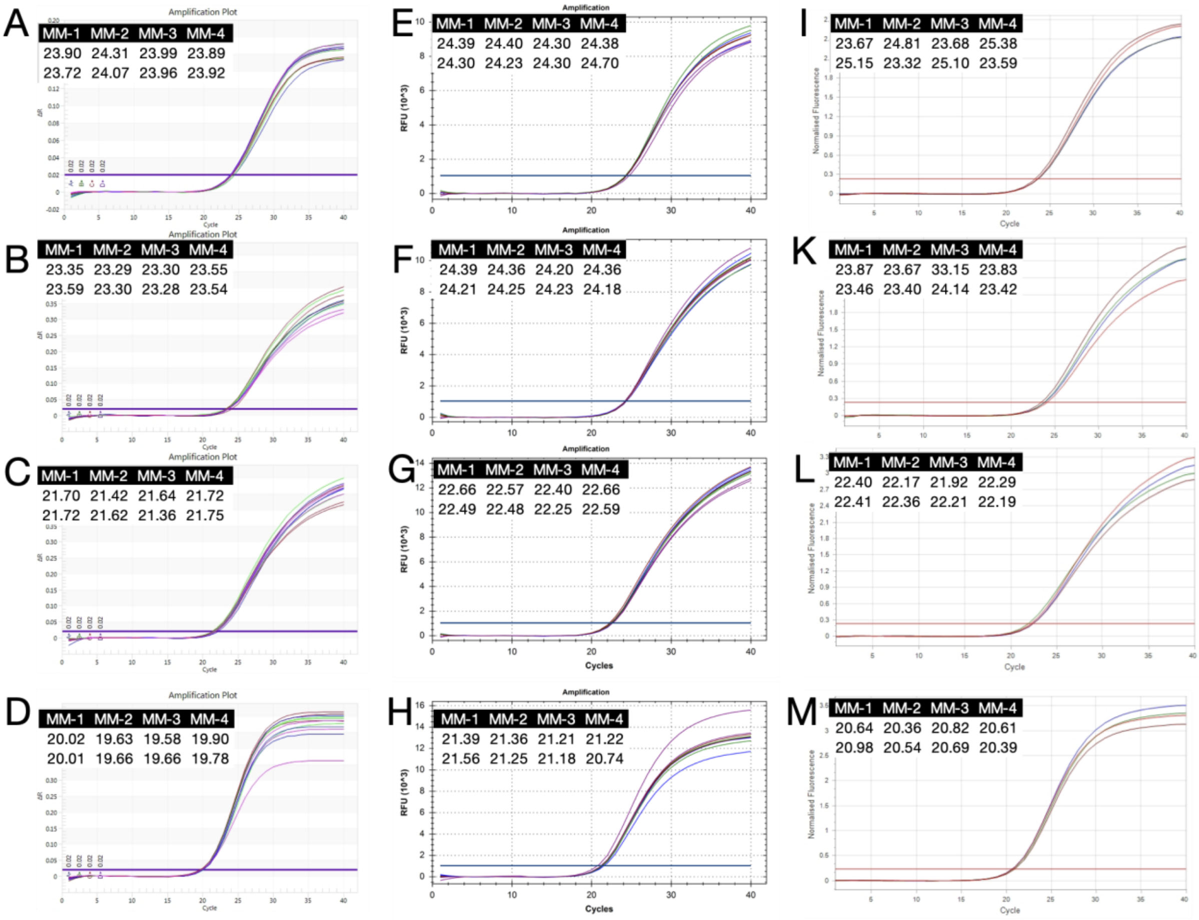

Figure S3. Comparison of "past expiry" and "in date" master mixes amplified on three different qPCR instruments. MM-1 (blue), MM-2 (green), MM-3 (brown), MM-4 (pink). **A-D.** Amplification plots and Cq values for assays A, B, C and D, respectively on a PCRMax Eco instrument. **E-H.** Amplification plots and Cq values for assays assays A, B, C and D, respectively on a BioRad CFX instrument. **I-M.** Amplification plots and Cq values for assays assays A, B, C and D, respectively on a BMS Mic qPCR cycler.

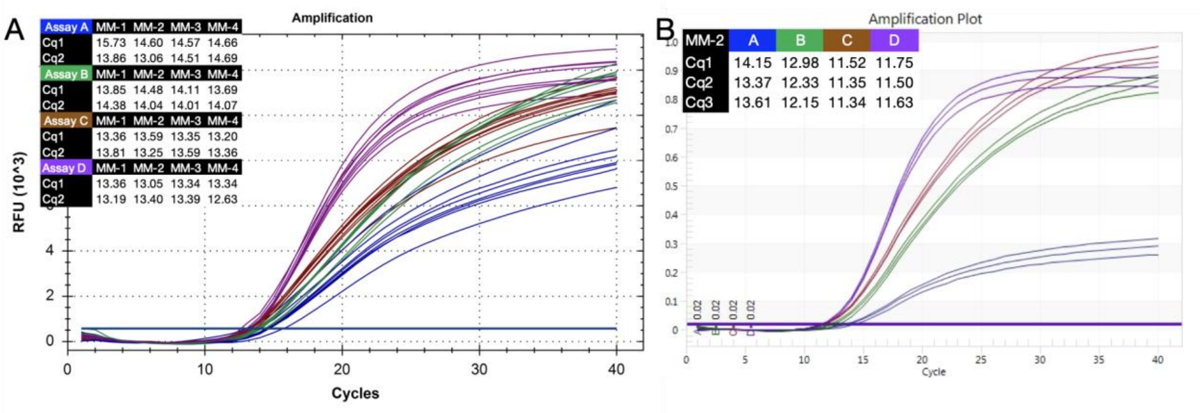

Figure S4. Comparison of "past expiry" and "in date" master mixes with high concentrations of target. Assay A (blue), Assay B (green), Assay C (brown), Assay D (purple). **A.** Amplification and detection on BioRad CFX instrument. **B.** Repeat of amplification and detection of the four targets with MM-2 on PCRMax Eco instrument.

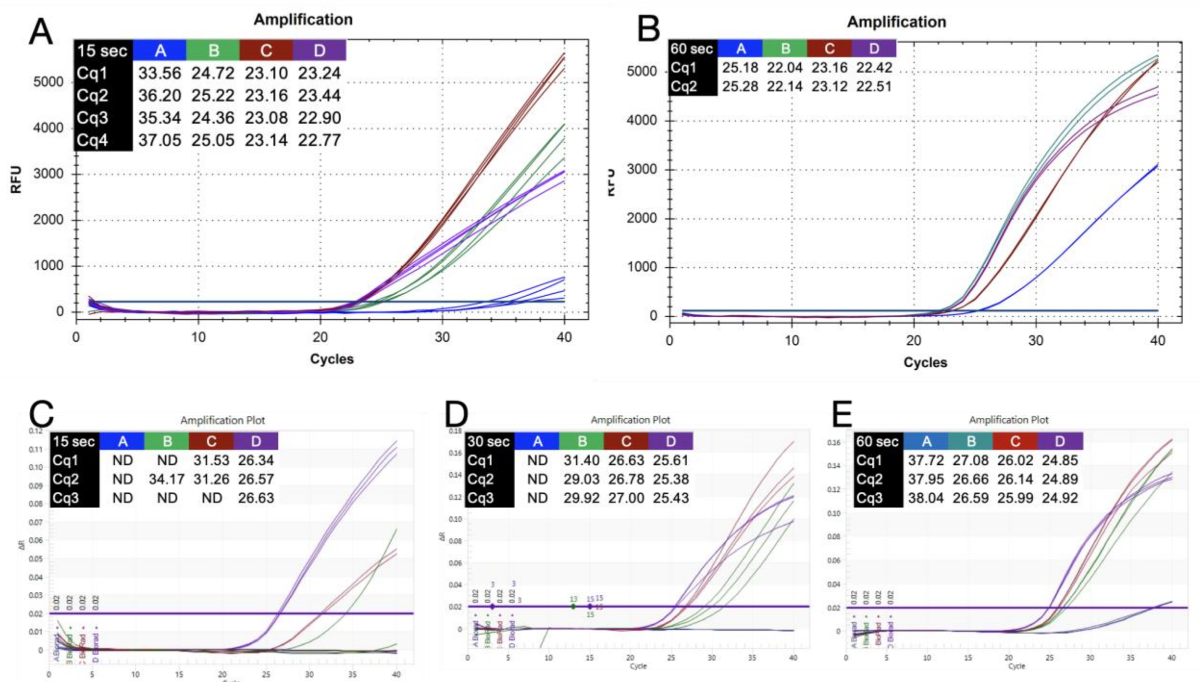

Figure S5. Analysis of BioRad master mix with assays A (blue), B (green), C (brown) and D (purple). **A.** Cq values and amplification plots recorded on the CFX instrument with a 15 second polymerisation time. **B.** Cq values and amplification plots recorded on the CFX instrument with a 60 second polymerisation time. **C.** Cq values and amplification plots recorded on the Eco instrument with a 15 second polymerisation time. **D.** Cq values and amplification plots recorded on the Eco instrument with a 30 second polymerisation time. **E.** Cq values and amplification plots recorded on the Eco instrument with a 60 second polymerisation time.

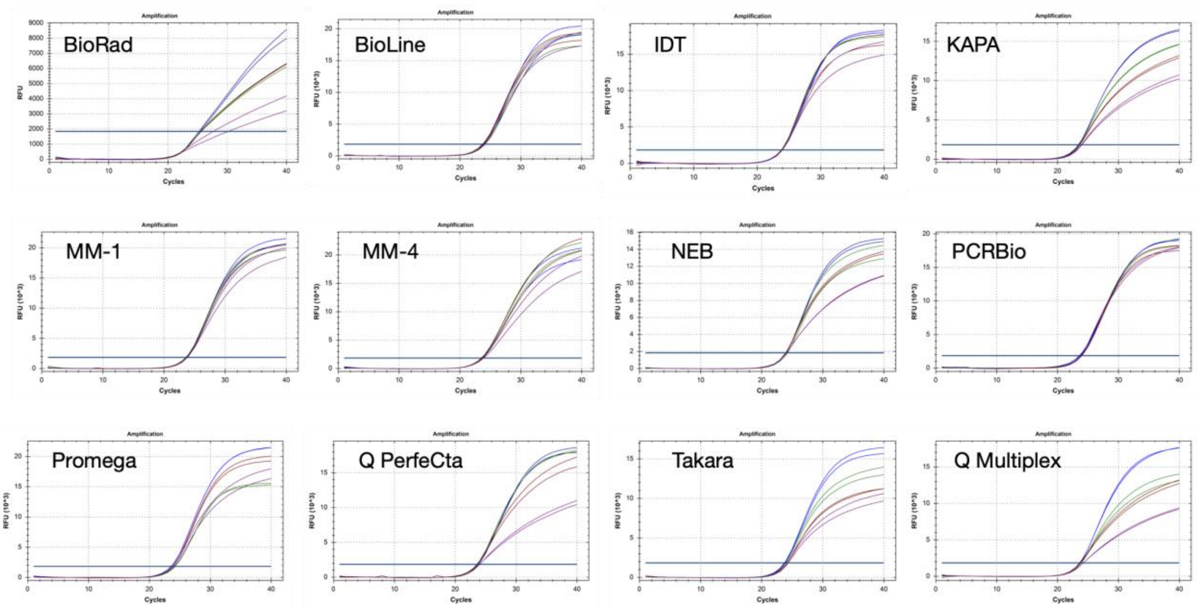

Figure S6. Amplification plots of assay D amplified by 12 master mixes at 1x (blue), 0.8x (green), 0.7x (brown) and 0.5x (purple) final concentrations.

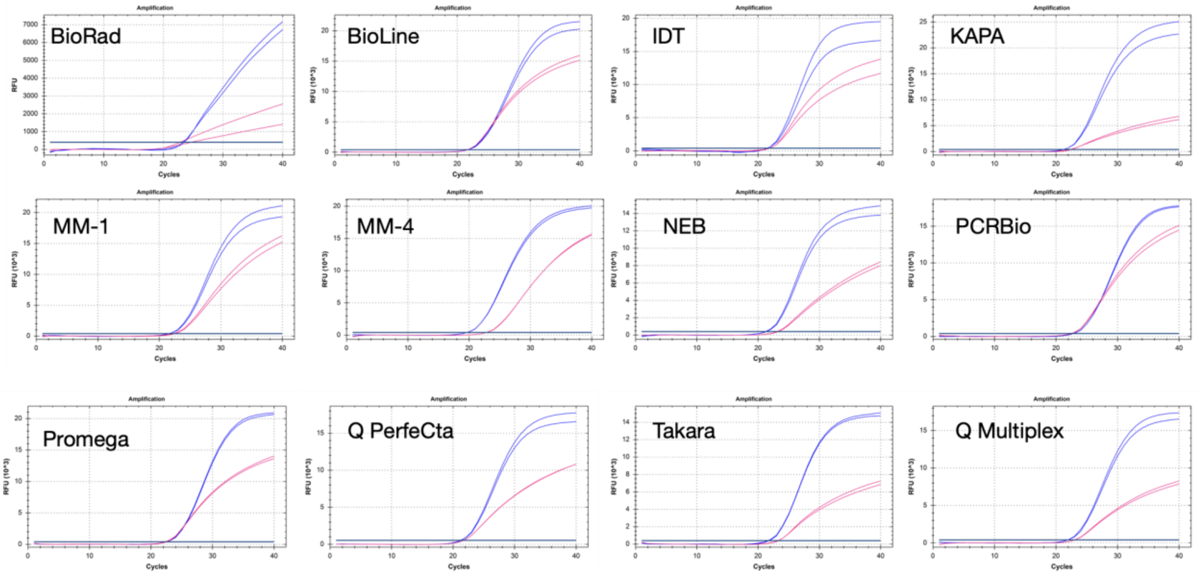

Figure S7. Repeat experiment showing amplification plots of assay D amplified by 12 master mixes at (1x (blue) and 0.4x (pink) final concentrations.
